# Quantitative Analysis of Metallographic Image Using Attention-Aware Deep Neural Networks

**DOI:** 10.3390/s21010043

**Published:** 2020-12-23

**Authors:** Yifei Xu, Yuewan Zhang, Meizi Zhang, Mian Wang, Wujiang Xu, Chaoyong Wang, Yan Sun, Pingping Wei

**Affiliations:** 1 School of Software, Xi’an Jiaotong University, Xi’an 710054, China; yuewan@stu.xjtu.edu.cn (Y.Z.); zhangmz99@stu.xjtu.edu.cn (M.Z.); alitongxue@stu.xjtu.edu.cn (M.W.); xjtuwujiangxu@stu.xjtu.edu.cn (W.X.); w1830768174@stu.xjtu.edu.cn (C.W.); sy0517@stu.xjtu.edu.cn (Y.S.); 2 State Key Laboratory for Manufacturing Systems Engineering, Xi’an Jiaotong University, Xi’an 710054, China; erin1989@xjtu.edu.cn

**Keywords:** metallographic analysis, image segmentation, object recognition, attention mechanism

## Abstract

As a detection tool to identify metal or alloy, metallographic quantitative analysis has received increasing attention for its ability to evaluate quality control and reveal mechanical properties. The detection procedure is mainly operated manually to locate and characterize the constitution in metallographic images. The automatic detection is still a challenge even with the emergence of several excellent models. Benefiting from the development of deep learning, with regard to two different metallurgical structural steel image datasets, we propose two attention-aware deep neural networks, Modified Attention U-Net (MAUNet) and Self-adaptive Attention-aware Soft Anchor-Point Detector (SASAPD), to identify structures and evaluate their performance. Specifically, in the case of analyzing single-phase metallographic image, MAUNet investigates the difference between low-frequency and high-frequency and prevents duplication of low-resolution information in skip connection used in an U-Net like structure, and incorporates spatial-channel attention module with the decoder to enhance interpretability of features. In the case of analyzing multi-phase metallographic image, SASAPD explores and ranks the importance of anchor points, forming soft-weighted samples in subsequent loss design, and self-adaptively evaluates the contributions of attention-aware pyramid features to assist in detecting elements in different sizes. Extensive experiments on the above two datasets demonstrate the superiority and effectiveness of our two deep neural networks compared to state-of-the-art models on different metrics.

## 1. Introduction

As a primary goal of metal science, physical or chemical properties are critical to inspect the quality of casting metal production. Among all the techniques of describing the properties, metallography has been widely used to reveal mesoscopic structural elements via the examination of metallurgical microscopes. Therefore, the quantitative analysis of metallographic images has achieved increasing attention to study the correlation between microstructure and metal properties. Generally, a steel microstructure is always a combination of different phases that refer to a physically homogeneous state of matter. Within an alloy, two and more different phases can be present, leading to irregular and complex substructures in metallographic image. In terms of single-phase metal image, accurate and effective segmentation results directly measure the quality and properties of given metal. In terms of the multi-phase metal image, only a fraction of the microstructure is taken into account to describe this correlation, so object detection is suitable to detect desirable constituents.

To date, in order to explore the correlation among metallographic images with single-phase or multi-phase, current metallography analysis still heavily relies on the advanced experts who evaluate a given picture of structure manually. Since the success of computer vision and image processing, we have witnessed their applications in face recognition, automatic driving, quantitative analysis of metal materials, and so on. In the past decades, enormous methods of image segmentation and object recognition have been developed to accomplish the above tasks efficiency. For image segmentation, the models roughly range from early rule-based and learning-based methods to recent deep-learning methods. The rule-based methods could offer accurate segmentation results, but often involve the prior rules, which greatly limit the generality in other applications [1,2]. The learning-based methods work based on handcrafted features, but they always suffer from the sensitively to constructed features for metallographic images with complex features [3,4,5]. Owing to the powerful ability of automatically learning the discriminable features, the recent surge of interest in deep learning methods has appeared in material science [6,7,8,9]. However, the major drawback of these methods is the poor ability of identifying microstructure instances. For object recognition, the current state-of-the-art object detectors are dominated by CNN-based algorithms. Both two-stage and one-stage detectors adopt region-based approaches to classify and local sampled regions [10,11,12,13,14,15,16]. Furthermore, to achieve better performance, most of them resort to Feature Pyramid Network (FPN) or multi-scale anchor boxers to explicitly handle objects with various size and shape. As an anchor box is associated with a certain level of feature map guided by handcrafted rules, these models are purely based on ad-hoc heuristics and unable to select the optimal feature level for each instance. To address the limitation, several anchor-free methods have been developed to assign each instance to the best feature level [17,18]. However, in metallographic practice, the data distribution of different components is biased and imbalanced, and the samples with similar appearance and shapes are difficult to be recognized. Namely, the issues of hard samples and imbalanced samples hinder the development of anchor-free detectors. To alleviate the problems, DeepMask [19], and RPN [12] rapidly narrow the number of candidates and object while filtering out background samples. Focal loss acts as a more effective loss for dealing with class imbalance and hard samples [17]. Nevertheless, they still assign equal or inaccurate weights to the training samples in the network design.

To address the above mentioned problems, we propose Modified Attention U-Net (MAUNet) and Self-adaptive Attention Soft Anchor-Point Detector(SASAPD) for analyzing metallographic images with single-phase and multi-phase, respectively. MAUNet, a reliable segmentation model based on U-Net and attention mechanism, puts emphasis on high-frequency loss during the connection used in an encoder-decoder network and introduces dual-path attention to improve the interpretability of features map at any resolution. SASAPD, a self-adaptive anchor-point detector based on SAPD, reranks and reweights the samples around the instance boxes to explicitly focus on hard samples, and assign optimal feature levels to given sample based on the loss distribution. For the pyramid features, light-weight attention modules are plugged in to boost detection accuracy. To verify the effectiveness of our proposed models, we conduct experiments on two metallographic datasets with single-phase and multi-phase, respectively. The experimental results demonstrate that our methods produce convincing results compared with state-of-the-art methods. Additionally, we make a series of ablation studies to verify the effectiveness of core components in our models.

In summary, our overall contributions are three-fold: (1) We propose MAUNet based on U-Net to segment single-phase metallographic images. The mentioned-above improvements allow our model to focus on the lost high-frequency information when transferring high-resolution information across the network, and enhance feature interpretability in decoders with the aid of spatial-channel attentions. (2) We propose SASAPD based on SAPD to detect constituents in multi-phase metallographic images. It improves soft-weighting scheme by reranking anchor points with powerful feature representation, and self-adaptively selects the reasonable features for each instance from attention-aware pyramid levels. (3) We conduct extensive experiments on metallographic images and compare with other state-of-the-art to figure out the superiority of our methods.

The rest of this paper is organized as follows. Section 2 displays the discussion of related work. In Section 3, the proposed method is described, and the experimental settings and evaluation metrics are described in Section 4. Section 5 presents the analysis and discussion of experimental results. Finally, Section 6 concludes the paper and suggests topics for future research.

## 2. Related Work

The topics of metallographic analysis are similar to those of general image segmentation and object detection. In this section, we will illustrate the related works of single-phase and multi-phase metallographic images using recent deep learning models.

With respect to the analysis of single-phase metallographic images, several researchers resort to CNN-based image segmentation methods and achieve significant performance [7]. 3D convolutional neural network [7] is proposed to extract microstructural properties. The 3DCNN is yet too heavy to apply in real-time application. Fully Convolution Networks (FCNs) have shown a lot of promise towards semantic segmentation [20]. The pioneers are DeepLab and its subsequent versions [21] which utilize atrous spatial pooling and multi-scale atrous pyramid features to enhance contextual information. However, these models fail to work well on the devices with limited computation resources, and require massive volumes of training data. Another line of works is encoder-decoder network, which combines deep, semantic, coarse-grained feature maps from the decoder with shallow, low-level, fine-grained feature map from the encoder. As a representative method, U-Net comprises an encoder and an decoder network which are connected by skip connection [22]. Owing to the low requirement of labeled training data, U-Net and U-Net like models have shown potential in different image segmentation application [23,24]. However, there are two obvious drawbacks when they are applied to metallographic image. One drawback is caused by the skip connection between low-level features and high-level features without enough high-frequency information, the other is the existence of irrelevant and redundant features, which prevents the interpretability of representative features in image segmentation. To address those drawbacks, we propose MAUNet with the assistance of extraction of high-frequency and dual-path attention module.

With regard to the analysis of multi-phase metallographic images, only a few works have been found to transfer classical object detector to recognize different constitutions [25]. Chen etc. use Mask R-CNN as the basic network to complete the learning and recognition of the latent feature of an aluminum alloy microstructure, but it suffers from the complex generation procedure of candidate proposals. In general, there are two main streams of object detection in the field of computer vision and image processing. As prevailing object detectors, anchor-based methods, which evolve from early proposal-based detectors, regard pre-defined proposals as priors for bounding box classification. They mainly include two branches for localization and classification: one-stage detector and two-stage detector. Recently, although a large number of anchor-based detectors have been developed [14,26]. The performance of anchor-based methods heavily depend on the pre-defined proposals. In most cases, the proposals are reluctant while ignoring the critical objects. Very recently, more and more attentions have been paid to anchor-free detectors. Instead of anchor boxes, the detectors based on keypoints locate several keypoints of the bounding boxes [27,28]. However, they have limitations such as relying on handcrafted clustering or post-processing steps to compose whole obvious objects from the detected points. Unlike keypoint-based detectors, anchor-point based detectors view a bounding box as an anchor point and its location. FCOS is an anchor-free detector to solve object detection in a per-pixel prediction fashion [29]. However, it treats all the sample equally, which cannot distinguish the positive and negative samples well. FSAF applies online feature selection to train anchor-free branches in the feature pyramid [18], but it only selects the optimal feature level for each instance. SAPD assigns optimal feature levels to given sample based on the loss distribution in object detection [30]. Whereas, it fail to obtain discriminable features due to the poor sample weighting strategy. AutoAssign [31] automatically determines positive/negative samples by generating positive and negative weight maps to modify each location’s prediction dynamically. Faced with the objects with similar appearances and shapes, AutoAssign fails to output satisfying results. Table 1 provides a summary of the related methods included in this study.

## 3. Methodology

In this section, we instantiate our two proposed models for image segmentation and object detection for metallographic images, respectively.

### 3.1. Network Structure of MAUNet

**Figure 1:**
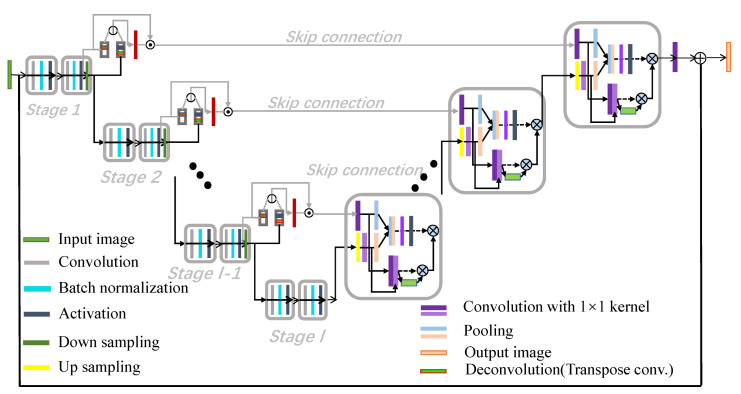
The architecture of Modified Attention U-Net (MAUNet). The proposed model has two main improvements, one is for the encoder part and the other is for the decoder.

Our proposed network is inspired by U-Net that captures feature information from encoders to decoders of similar resolutions. The architecture of our proposed MAUNet is depicted in Figure 1. Compared with original U-Net network [32,33], we mainly contribute three points to boost the segmentation performance on metallographic images. (1) For each encoder, high-frequency is extracted and transferred with skip connection to prevent smoothing the object boundary information in segmentation result. (2) For each decoder, a dual-path attention block is proposed to yield strong results with inherent interpretability, and give importance to a certain region out of the entire image. (3) The overlap tile strategy is ignored to reduce the effects of overlapped results, and Batch Normalization (BN) is added to speed up network training.

In our work, we keep the basic architecture of U-Net, and make improvements on the encoders and decoders. In convolutional U-Net, skip connections between encoders and decoders are utilized to pass high-resolution information throughout the network. In this way, only the low-frequency information filtered by pooling operations passes on to the next encoder while the high-frequency information is lost. As reported in [34,35], the low-frequency duplication in U-Net will lead to the missing high-frequency information. To avoid it, skip connection is employed to ensure that U-Net preserves the full context of the input images. However, the low-frequency information goes along with this skip connection as well, which will always smooth object boundary. Therefore, we design a frequency-aware encoder (FAE) to transfer high-frequency information with convolutional skip connection. Let $O^{l-1}$ and $D^{l-1}$ denote the outputs before and after the last downsampling layer of stage (l−1). We first adopt two dilated convolutions to extract features in different receptive fields. The two convolutions $f_{d1}$ and $f_{d2}$ are kernel=1 with dilation rate =3 and kernel=1 with dilation rate =3, respectively. Then, we consider a high-frequency ratio map $r_{l-1}$ between these two groups.

(1) $$r_{l-1}=sigmoid\left(f_{d1}\left(O^{l-1}\right)-f_{d2}\left(UP\left(D^{l-1}\right)\right)\right)$$

where UP(·) is upsampling layer and sigmoid is sigmoid function. Lastly, we multiply $O_{l-1}$ by $r_{l-1}$ to obtain high-frequency map $H_{l-1}=O_{l-1}\cdot r_{l-1}$. In skip connection, we append a block of convolution layers to provide enough high-frequency content for higher level feature maps.

**Figure 2:**
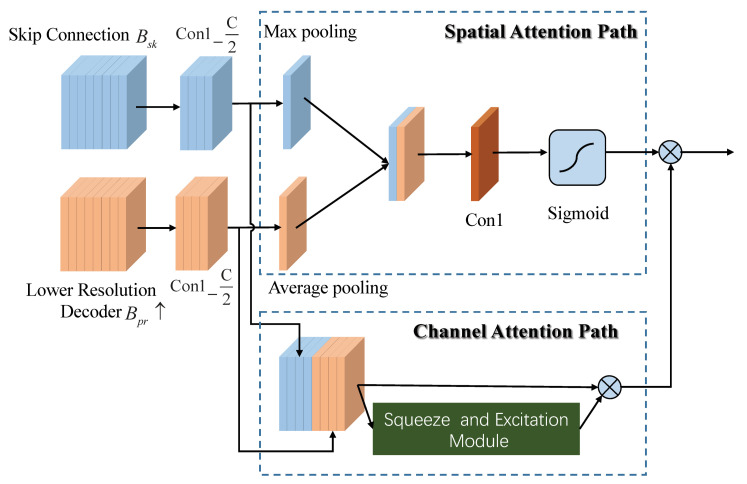
The flowchart of dual-path attention models.

In convolutional U-net, the decoder fuses feature maps from FAE with skip connection along with the feature maps from lower-resolution decoder. In order to increase feature representation power, we propose dual-path attention models by blending cross-channel and spatial information together. Now, as illustrated in Figure 2, we will detail the two attention models as follows. (1) Spatial attention path. Inspired by [36], we use max-pooling and average-pooling along the channel axis to extract spatial attention. For the branch of FAE with skip connection $B_{sk}$ with *C* channels, max-pooling operation and $1\times 1\times \frac{C}{2}$ convolution are applied to generate the feature descriptor denoted by $F_{max}^{s}$. For the branch of lower resolution decoder $B_{pr}$ with *C* channels, average-pooling operation and $1\times 1\times \frac{C}{2}$ are applied to compute the average statistics of all channels denoted by $F_{avg}^{s}$. The above $F_{max}^{s}$ and $F_{avg}^{s}$ are concatenated and forwarded to a 1×1 convolution layer followed by a sigmoid function, generating spatial attention map $F^{s}$. Besides, as described in Equation (2), the concatenation of $Con1\_\frac{1}{C}\left(B_{sk}\right)$ and $Con1\_\frac{1}{C}\left(B_{pr}\right)$ is scaled by $F^{s}$ to obtain spatial-aware feature map $F^{s^{\prime }}$.

(2) $$\begin{array}{c} F_{max}^{s}=max-p\left(Con1\_\frac{C}{2}\left(B_{sk}\right)\right)\\ F_{avg}^{s}=avg-p\left(Con1\_\frac{C}{2}\left(B_{pr}\right)\right)\\ F^{s}=sigmoid\left(Con1\left(concat\left(F_{max}^{s},F_{avg}^{s}\right)\right)\right)\\ F^{s^{\prime }}=Concat\left(Con1\_\frac{1}{C}\left(B_{sk}\right),con1\_\frac{1}{C}\left(B_{pr}\right)\right)⊗F^{s} \end{array}$$

where Concat denotes concatenation operation, max-p(·) and avg-p(·) are max-pooling and average-pooling, respectively. $Con1\_\frac{C}{2}$ is the convolution with $1\times 1\times \frac{C}{2}$ and ⊗ is element-wise multiplication. (2) Channel attention path. As suggested in [36], we replace global average-pooling with max-pooling in Squeeze-and-Excitation to infer fine channel attention $F_{c}$. With the output of spatial-ware feature map $F^{s^{\prime }}$, the channel and spatial attention map $F_{c}^{s}$ can be written as $F_{c}^{s}=F^{s^{\prime }}⊗F_{c}$.

Apart from the above-mentioned improvements, U-net is first proposed to handle segmentation problem in medical image processing. In our case, the resolution of metallographic image is less than the one of medical image. Hence, we don’t decide to take the overlap title strategy into consideration. Besides, BN is added to speed up the training procedure in our model. The learning process of MANU is illustrated in Algorithm 1.
**Algorithm 1** The learning process of MAUNet**Input:** The training images $I_{train}$, max-epochs E=12, the number of $I_{train}$$N_{train}$, The testing image $I_{test}$ and the groundtruth labels *G***Output:** The output prediction $G^{{}^{\prime }}$, and its performance results $G_{Dice}^{{}^{\prime }}$, $G_{IoU}^{{}^{\prime }}$, $G_{RoC}^{{}^{\prime }}$ and $G_{time}^{{}^{\prime }}$ All the images are preprocessed according to the steps in Section 4.2. ***Training Stage*:**
 Initialize the network weights, learning rate, batch size, and other parameters **for**
i=1; i≤E; i++**do**  Get the data batch from $I_{train}$
  **for**
j=1; $j\leq N_{train}$; j++
**do**
   Compute IoU loss function $L_{IoU}$;
   Compute Dice loss $L_{Dice}$;
   Compute Focal loss $L_{Focal}$;
   Train MAUNet by optimizing loss $L_{MAUNet}$ and update the weights and parameters;
**  end for**** end for***** Testing Stage:***
 Feed $I_{test}$ into the well-trained MAUNet and then output the prediction segmentation $G^{{}^{\prime }}$;

 Compute the performance results $G_{Dice}^{{}^{\prime }}$ (Equation (14)), $G_{IoU}^{{}^{\prime }}$ (Equation (15)), $G_{RoC}^{{}^{\prime }}$ (Equation (16)) and running time $G_{time}^{{}^{\prime }}$
** return**$G^{{}^{\prime }}$, $G_{Dice}^{{}^{\prime }}$, $G_{IoU}^{{}^{\prime }}$, $G_{RoC}^{{}^{\prime }}$ and $G_{time}^{{}^{\prime }}$.


### 3.2. Hybrid Loss for MAUNet

Aside from network architecture, loss function also plays a key part in network design. It often measures the similarity between the ground-truth and predicted result. In this section, we present a hybrid loss as follows:

(3) $$L_{MAUNet}=L_{IoU}+0.01L_{Dice}+0.8L_{Focal}$$

where $L_{IoU}$ is the IoU loss optimized for segmentation mismatch error [37]. $L_{Dice}$ measures the overlap and similarity between prediction and ground-truth labels [38], and $L_{Focal}$ is introduced to solve the problem of serious imbalance between positive and negative samples [17]. Intuitively, the model learns to predict individual pixel values correctly through $L_{Focal}$ and $L_{IoU}$, and also learns to consider overlap through $L_{Dice}$. Here, the settings of those three weights are referred to [39].

### 3.3. Network Structure and Loss of SASAPD

**Figure 3:**
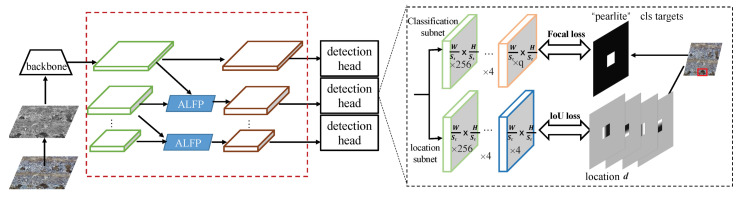
The network architecture of self-adaptively attention-aware soft anchor-point detector.

In this section, we propose a Self-adaptive Attention-aware Soft Anchor-Point Detector (SASAPD) to detect the constitutions in multi-phase metallographic images. In Figure 3, we present the architecture of SASAPD which almost has the same structure as SAPD except the part of the pyramid levels. Similar to SAPD, it aims at solving the tasks of classification and location simultaneously. The classification subset is designed to obtain the probability of each anchor point of q(q=5) object classes, and the location subnet predicts 4-dimensional location of each anchor point when it is positive. Inspired by SAPD and attention mechanism, our proposed model mainly makes the following improvements: (1) A new Sampling Reweighting Strategy (SRS) is designed to prevent attention bias, which is an effective way of perceiving the constitution in smaller size. (2) A Soft Self-adaptive Selection (3S) strategy is proposed to get rid of the reliance on pre-trained one-hot vector indicating the pyramid level with minimal loss. (3) Attention blocks are integrated into pyramid-level features to focus on the locations related for target constitution. The details of the above improvements are listed as follows. During the process of multi-phase metallographic structures, the performance of traditional object detector is degraded due to dirty spots and similar appearance, and it can be ascribed to the attention bias problem.

**Figure 4:**
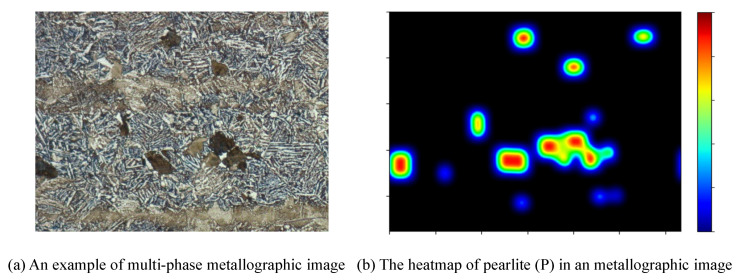
The visualization of the attention bias of pearlite (P) in a multi-phase metallograph.

In Figure 4, we visualize the attention bias of pearlite (P) in a multi-phase metallographic image. As can be seen, the pearlite (P) with larger size gets higher response in the heatmap, and it tends to expand towards the other underrepresented areas. In practice, attention bias will cause attention to the obvious areas while ignores the others with insufficient features. To tackle this difficulty, SAPD assigns weight for each point depending on the distance between its location and the corresponding instance center. However, given two anchor points with the same distance but with different locations, SAPD will assign the same weight in spite that they make different contributions to final loss. Now, let us revisit the influence of the true positives and true negatives. We define a ground-truth instance box B=(c,x,y,w,h) and its central shrunk box $B^{v}=\left(c,x,y,\epsilon w,\epsilon h\right)$, where *c* is class id, (x,y) is the box center, and *w*, *h*, ϵ are the box width, height and the shrunk factor, respectively. Given an anchor point $p_{l_{ij}}$ with predicted class $c_{l_{ij}}$ inside instance box $B{\left(k\right)}_{l}$ appeared in pyramid level *l*, it will be marked as true positive (TP) if $c_{l_{ij}}=={\hat{c}}_{l_{ij}}$, otherwise it is true negative (TN) when $c_{l_{ij}}\neq {\hat{c}}_{l_{ij}}$. In our case, TN is much less important than TP since TN is easily discarded after Non-Maximum Suppression(NMS). Among all the TP anchor points, the one with the highest IoU has the greatest impact as it directly affects the performance precision and recall. Moreover, for the negative anchor points, they only involve the classification procedure, which can be balanced with the following focal loss. To overcome the above shortcoming, we propose SRS to rerank and reweight the true samples. For each shrunk instance $B^{v}{\left(k\right)}_{l}$, we descend the anchor points $p_{l_{ij}}$ according to the following score.

(4) $$score=|sign\left(c_{l_{ij}}-{\hat{c}}_{l_{ij}}\right)|\left(1-\alpha \right)IoU\left(d_{l_{ij}},{\hat{d}}_{l_{ij}}\right)$$

where sign(·) denotes symbol function and α is the modulating factor and set to 0.1. Here, $d_{l_{ij}}$ and ${\hat{d}}_{l_{ij}}$ indicate the ground-truth location and predicted location, respectively. With SRS strategy, we assign lower scores to TN examples which are less important than TP ones. For instance, for box $B^{v}\left(k\right)$, its size is $n{\left(k\right)}_{v}=area\left(B^{v}\left(k\right)\right)$, with the above resultant scores, we compute the collection $f(p_{l_{ij}})$ that maps anchor point $p_{l_{ij}}$ to the ranking indexes $1,2,\cdots ,n_{max}$ where $n_{max}$ denotes the maximum value of $n{\left(k\right)}_{v}$ over all the instance boxes. Also, given instance set $UB=B^{v}{\left(1\right)}_{l}\cup B^{v}{\left(2\right)}_{l}\cdots \cup B^{v}{\left(k\right)}_{l}\cdots B^{v}{\left(N\right)}_{l}$ in an image, the weight of $p_{l_{ij}}$ in UB is formulated as follows:

(5) $$w_{l_{ij}}=\left\{\begin{array}{cc} 1-\frac{f(p_{l_{ij}})}{n_{max}} & \;p_{l_{ij}}\in UB\\ 1 & \;otherwise \end{array}\right.$$

The anchor-free idea allows us to learn informative representation from an arbitrary pyramid-level. Unlike selecting feature depending on box size like FPN [40], we borrow the idea from FASF [18] and Foveabox [41]. As mentioned in SPAD, the contributions of multiple feature levels rely on the pattern of feature response. Therefore, we propose 3S strategy to reweight the pyramid levels of each instance. A weight is assigned to each pyramid level according to the feature response, which can be viewed as assigning a proportion of the instance to a level. As FoveaBox [41] suggests, assigning instances to multiple but not all pyramid levels can boost the performance. So, the 3S strategy only assigns instance to top k(k=3) pyramid levels, and determines the weights of each pyramid level by evaluating the loss defined in Equation (7). For anchor point $p_{l_{ij}}$, its ground-truth and predicted pair (class id, location) are written as $v_{l_{ij}}=\left(c_{l_{ij}},d_{l_{ij}}\right)$ and ${\hat{v}}_{l_{ij}}=\left({\hat{c}}_{l_{ij}},{\hat{d}}_{l_{ij}}\right)$, respectively. For instance, for $B^{v}{\left(k\right)}_{l}$, the per anchor point loss $L_{l_{ij}}$ is written as

(6) $$L_{l_{ij}}=\left\{\begin{array}{cc} L_{FL}\left(c_{l_{ij}},{\hat{c}}_{l_{ij}}\right)+L_{IoU}\left(d_{l_{ij}},{\hat{d}}_{l_{ij}}\right) & p_{l_{ij}}\in B^{v}{\left(k\right)}_{l}\\ L_{FL}\left({\hat{c}}_{l_{ij}},c_{l_{ij}}\right) & otherwise \end{array}\right.$$

The loss for pyramid level *l* is the sum of all anchor point losses. For all the pyramid levels, we calculate loss $w_{l_{ij}}L_{l_{ij}}$ for each level, and define the weights for instance box $B^{v}{\left(k\right)}_{l}$ that acts as a good reflection of distinct contributions.

(7) $$L_{l}=\frac{1}{area(B^{v}{\left(k\right)}_{l})}\sum_{i,j}L_{lij}$$

(8) $$w_{l}=\left(1-\sigma \right)\frac{L_{l}}{\sum L_{l}}+\sigma$$

where σ represents the minimum sample weight and is set to 0. With the pyramid level weight $w_{l}$, Equations (5) and (7) are augmented into Equations (9) and (10), respectively.

(9) $$w_{l_{ij}}=\left\{\begin{array}{cc} w_{l}\left(1-\frac{f(p_{l_{ij}})}{n_{max}}\right) & \;p_{l_{ij}}\in UB\\ 1 & \;otherwise \end{array}\right.$$

(10) $$L=\frac{1}{\sum_{p_{l_{ij}}\in p_{+}}w_{l_{ij}}}\sum w_{l_{ij}}L_{l_{ij}}$$

where $p^{+}$ is the set of positive anchor points.

**Figure 5:**
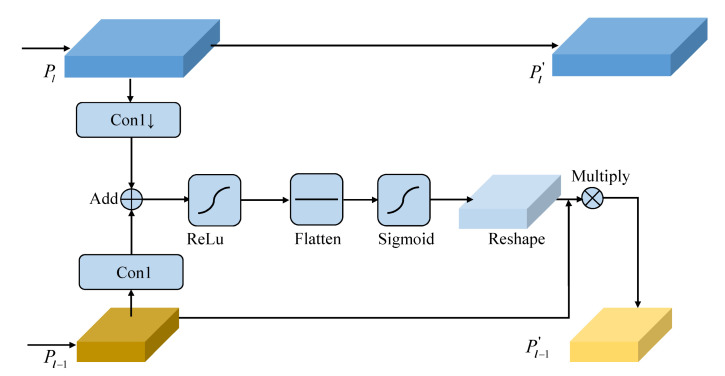
The procedure of attention-aware lightweight feature pyramid (ALFP).

A good attention module can help the detector to perceive indistinguishable constitution, such as steel element in multiple-phase metallographic images. To implement this, we replace the above pyramid level with an attention-aware lightweight feature pyramid (ALFP). In Figure 5, we consider the context between adjacent feature maps and then use an attention map to increase the receptive ability of pyramid features. In detail, a pyramid level is denoted as $P_{l}$ where *l* is the level number and it has $\frac{1}{s_{l}}$ resolution of the input image. $s_{l}$ is the feature stride and $s_{l}=2^{l}$. At first, two pyramid levels $P_{l}$ and $P_{l-1}$ are all passed through 1×1 convolutions to align along the channel dimension. Then, $Con1(P_{l})$ is downsampled ×2 and then concatenated by $Con1(P_{l-1})$ followed by ReLu activation function.

(11) $$C_{l-1}=ReLu\left(Con1\left(P_{l}\right)↓⊕Con1\left(P_{l-1}\right)\right)$$

where Con1 refers to 1×1 convolution and ⊕ refers to addition operation. In order to exploit the context information from adjacent levels, we flatten $C_{l-1}$ followed by sigmoid function and then reshape it to the original size of $C_{l-1}$. The $P_{l-1}^{{}^{\prime }}$ is obtained by element-wise multiply $reshape(sig\left(flat\left(C_{l-1}\right)\right))$ with $P_{l-1}$. To sum up, the learning process of SASAPD is placed in Algorithm 2.

(12) $$P_{l-1}^{{}^{\prime }}=reshape\left(sig\left(flat\left(C_{l-1}\right)\right)\right)⊗P_{l-1}$$

**Algorithm 2** The learning process of SASAPD**Input:** The training images $I_{train}$, max-epochs E=12, the number of $I_{train}$
$N_{train}$, The testing image $I_{test}$ and the groundtruth labels *G*
**Output:** The output prediction $G^{{}^{\prime }}$, and its performance results $G_{Dice}^{{}^{\prime }}$, $G_{Recall}^{{}^{\prime }}$, $G_{Precision}^{{}^{\prime }}$ and $G_{FPS}^{{}^{\prime }}$
 All the images are preprocessed according to the steps in Section 4.2.
 ***Training Stage:***
 Initialize the network weights, learning rate, batch size, and other parameters;
 **for**i=1; t≤6; i++
**do**
  **for**
j=1; $j\leq N_{train}$; j++
**do**
   Get the data batch from $I_{train}$
   Compute loss function $L_{l}$ (Equation (7))
   Each instance is assigned to the pyramid level which has the minimal loss $L_{l}$
   Train SASAPD by optimizing loss L (Equation (10)) where $w_{l}=1$, and update the weights and parameters;
  **end for**
 **end for**
 **for**
i=7; i≤E; i++
**do**
  **for**
j=1; $j\leq N_{train}$; j++
**do**
   Get the data batch from $I_{train}$
   Compute loss function $L_{l}$ (Equation (7))
   Train SASAPD by optimizing loss L (Equation (10)), and update the weights and parameters;
  **end for**
 **end for**
 Feed $I_{test}$ into the well-trained SASAPD and then output the prediction segmentation $G^{{}^{\prime }}$;
 Compute the performance results $G_{Dice}^{{}^{\prime }}$ (Equation (14)), $G_{Recall}^{{}^{\prime }}$ (Equation (17)), $G_{Precision}^{{}^{\prime }}$ (Equation (17)) and running time $G_{FPS}^{{}^{\prime }}$
 **return**
$G_{Dice}^{{}^{\prime }}$, $G_{IoU}^{{}^{\prime }}$, $G_{RoC}^{{}^{\prime }}$ and $G_{FPS}^{{}^{\prime }}$.



## 4. Experiments

### 4.1. Dataset and Data Preparation

**Figure 6:**
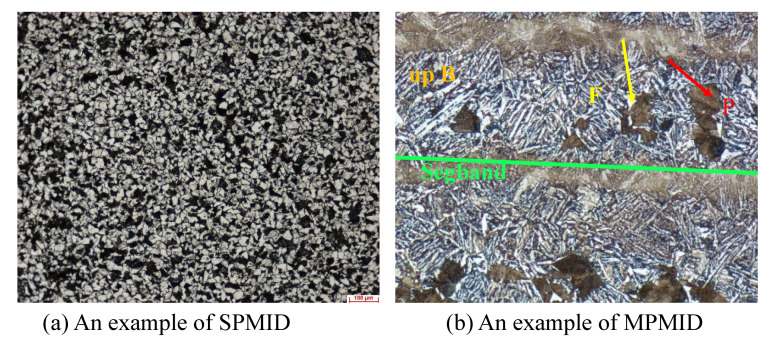
The examples of our built datasets. (**a**) SPMID (**b**) MPMID.

To facilitate the learning of the proposed models, we have prepared two new metallographic image datasets that cover single-phase and multi-phase types. All the images are collected and built with Zeiss intelligent microscope Axio Imager A2m under the optical microscopy 100× magnification. In terms of Single-Phase Metallographic Image Dataset (SPMID), we treat the microscopic images of cross section of hot rolled section steel 20G as our observations. Based on carbon level (level A~E) and segregation degree (degree 1~5), we illustrate the distribution of all the samples in Table 2. Through the segmentation results of SPMID, we can explore the roundness of MC-type carbide, which could be used for quality evaluation of steel production. The dataset includes 7500 training images, 500 validating images and 1433 testing images. In view of Multi-Phase Metallographic Image Dataset (MPMID), we build it on the longitudinal section of round steel 42CrMo. As shown in Figure 6b, the sample contains a series of structures such as upper bainite (up B), ferrite (F), pearlite (P) and segregation band (Segband). Apparently, the sizes of those objects have large differences. For each sample, Segband is much larger than up B. We annotate all the structures by drawing an bounding box around target object with annotation tool-LabelMe. With the well-trained detector, we can easily evaluate steel quality by calculating the statistics of constitutions. To prevent overfitting, horizontal image flipping is utilized in data augmentation. In total, we use 6500 metallographic images for training, 500 images for validating and the remaining 1000 for testing. In Table 3, we count the proportions of F, P, up B, Segband and background in dataset MPMID. As can be seen, the distribution of data samples is biased and imbalanced. The F elements make up nearly 41.8% while the up B elements are in the minority.

### 4.2. Dataset Preprocessing

**Figure 7:**
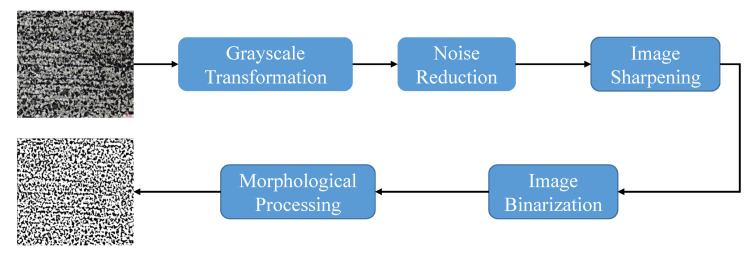
The pipeline of image preprocessing for morphological images

With the purpose of obtaining better result, it is necessary to carry out preprocessing procedure for removing noise and enhancing quality. As illustrated in Figure 7, we apply a series of operations to the above two datasets. (1) Grayscale transformation. To enhance image quality and reduce computation cost, as a preliminary step, the metallography in RGB is transformed to grayscale format. (2) Noise elimination. During acquiring metallographic samples, there are undesirable noise and digital artifacts caused by microscope equipment and operating environment. With the presence of noise, the subsequent image processing tasks, image segmentation or object detection, are adversely affected. As an effective and efficient method, Robust PCA has been widely used for denoising in different fields [42,43]. In fact, RPCA (Robust PCA) only works well when the noise is in accordance with sparse and low-rank representation. If we directly apply it to our datasets, the subtle microstructures will be removed. Thus, we propose Patch-constrained Robust Principal Component Analysis (PRPCA) denoising algorithm which adds patch constraint to the following objective function.

(13) $$\underset{A,E}{min}\left({∥A∥}_{*}+\lambda {\left|E\right|}_{1}+\gamma \sum_{i=1}^{n}{∥A_{i}∥}_{2}^{2}\right),\;s.t.A+E=D$$

where ${∥\cdot ∥}_{*}$ is nuclear norm. ${∥\cdot ∥}_{1}$ and ${∥\cdot ∥}_{2}$ are $l_{1}$ norm and $l_{2}$ norm, respectively. Parameters λ and γ are weight coefficients. *A* and *E* stand for clean image and additional noise, respectively. $D\in R^{m\times n}$ is noisy image. $A_{i}$ denotes the *i*th patch of image *A* with size of $\frac{m}{4}\times \frac{n}{4}$ where *m* and *n* are the width and height of image *A*, respectively. After denoised by RPCA, nearly clean images can be obtained. (3) Image sharpening. Image sharpening is a technique for enhancing fine details and edges. We use Butterworth high filter with 4th order and cut-off lower frequency to improve image quality. (4) Image binarization. Image binarization is the process of taking a grayscale image and converting it to black-and-white. In our step, Sauvola binarization is applied to dataset SPMID with ill illumination. Note that the images in MPMID are not involved as the detection accuracy heavily depends on gray change and object details. (5) Morphological processing. Morphological processing pursues the goal of removing imperfections. After a combination of erosion, dilation and simple set-theoretic operations, imperfections are eliminated and image quality is improved. When finishing the above operations, as shown in Figure 7, we see that image quality is obviously improved.

### 4.3. Performance Evaluation Metrics

#### 4.3.1. Evaluation Metrics for Segmentation

In this section, we first introduce two popular overlap-based metrics to evaluate the performance quantitatively. Dice coefficient (Dice) is double the area of overlap divided by the total number of pixels in both image samples. It ranges from 0 to 1 and could be described as:

(14) $$Dice=\frac{2|G\cap G^{\prime }|}{\left|G|+\right|G^{{}^{\prime }}|}$$

where Dice with value 1 denotes perfect and complete overlap. *G* and $G^{{}^{\prime }}$ are the ground-truth and predicated segmentation, respectively. Herein, another common-used metric named IoU is introduced and calculated as:

(15) $$IoU=\frac{|G\cap G^{\prime }|}{\left|G|+\right|G^{{}^{\prime }}|-|G\cap G^{{}^{\prime }}|}$$

As we all know in metallographic science, the roundness of carbide is closely relevant to the steel quality. So, we introduce a specific metric for our dataset-Roundness of Carbides (RoC). RoC is crucial to evaluate the steel quality. The diameter is easily acquired by Image-Pro Plus 2D image analysis software. In most cases, the more rounded the carbide is, the better is the steel. Mathematically, RoC is formulated as

(16) $$RoC=\frac{4\pi S}{L^{2}}$$

where *S* and *L* are the size and diameter of a given carbide tissue.

#### 4.3.2. Evaluation Metrics for Object Detection

It is an important issue to evaluate the performance of the proposed method on multi-phase dataset. At present, there are three main performance evaluation metrics: Precision, Recall and Dice. Dice has been present in Equation (14) where *G* and $G^{{}^{\prime }}$ mean the ground-truth and predicted bounding box, respectively. Precision and Recall are based on the statistical True Positives (TP), False Positives (FP), True Negatives (TN) and False Negatives (FN). Here, whether the predicted $G^{{}^{\prime }}$ is correct or not is determined by the IoU threshold. In our experiments, when IoU>0.5, the result is considered correct Otherwise, it is wrong. Therefore, the Precision and Recall of each class can be computed as:

(17) $$\begin{array}{c} Precision=\frac{TP_{C_{ij}}}{TP_{C_{ij}}+FP_{C_{ij}}}\\ Recall=\frac{TP_{C_{ij}}}{TP_{C_{ij}}+FN_{C_{ij}}} \end{array}$$

where $C_{ij}$ represents class $C_{i}$ of the *j*th image. In actual scene, the grain size plays a critical role in estimating the steel quality. For simplicity, we can use metric Recall as the measure of grain size.

### 4.4. Learning Parameters and Training Details

Our experiments are implemented in Pytorch and performed on a ${\mathrm{NVIDIA}}^{®}$ Tesla P100 GPU by optimizing the loss mentioned in Section 3.2 and Section 3.3. All the models are trained by the Adam optimizer with $\beta_{1}=0.9$ and $\beta_{2}=0.999$ along with weight decay of $1\times 10^{-4}$. The initial learning rate of $7\times 10^{-4}$ exponentially decayed with parameter 0.99. At the step of image preprocessing, λ and γ are set to $\frac{1}{\sqrt{max(m,n)}}$ and $\frac{1}{ceil(\sqrt{max(m,n)}/t)}$, respectively where ceil is the ceiling fnctuion. In terms of MAUNet, the input images are resized to 512×512 to reduce computation cost. The stage of Figure 1 is set to 5, and it is trained for 12 epochs with a batch size of 4. The base hyperparameters of the networks is consistent with original U-Net. In terms of SASAPD, the input size is 224×224 which is compatible with pre-trained ResNeXt-101 network [44]. The number of pyramid level is set to 5. The classification layers in detection head are initialized with bias −log((1−π)/π) where π=0.01 and a Gaussian weight filled with σ=0.01. The location layers in the detection head are initialized with bias 0.1 and a Gaussian weight filled with σ=0.01. The shrunk factor ϵ is set to 0.2. In order to stabilize the training model, at the first 6 epochs, each instance is assigned to the pyramid level which has the minimal loss. For the next 6 epochs, 3S strategy is adopted to reweight the contributions of pyramid features. All relevant codes will be available in https://github.com/ZhangYuewan/Metallographic-Image-Analysis.

### 4.5. Experiments on Dataset SPMID

**Figure 8:**
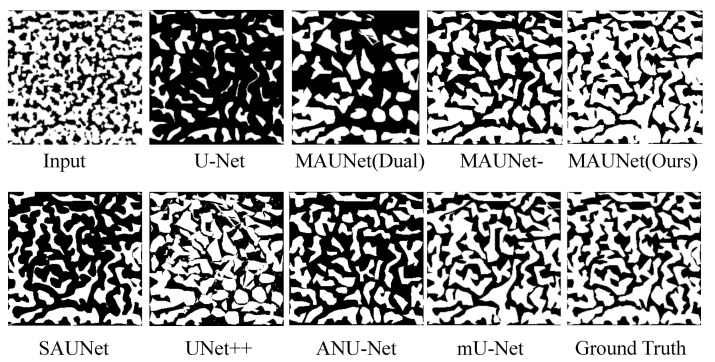
The visual comparison of the segmentation results on dataset SPMID.

**Figure 9:**
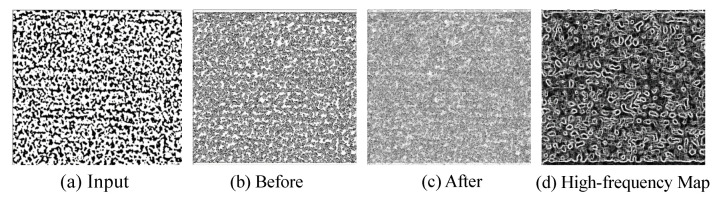
The visual feature maps and the high-frequency map. (**a**) Input image (**b**) The feature before the last downsampling layer (**c**) The feature after the last downsampling layer (**d**) High-frequency map.

To clarify the comparison, we conduct several experiments to verify the superiority of MAUNet on the task of image segmentation. The experiment includes two parts: The first part make ablation study of our framework, and the second part evaluates our proposed framework against several state-of-the-art methods quantitatively and qualitatively. The proposed MAUNet evolves from U-Net network, so we choose U-Net as our baseline. To further verify the effectiveness of each part, we have done the following experiments on dataset SPMID comprehensively. (1) UNet: It is trained with the above-mentioned parameters and hyperparameters. (2) MAUNet(Dual): Compared with U-Net, it only replaces the decoder with our dual-path attention module and keep the rest unchanged. (3) MAUNet-: Compared with MAUNet, the overlapped strategy is adopted and BN layer is removed. Besides, we quantitatively and visually compare our model with several state-of-the-art segmentation models, including mU-Net [45], UNet++ [46], ANU-Net [47], SAUNet [48] and Deeplab V3+ [49]. Besides, we also visualize the feature maps before and after the last downsampling layer of stage 1. The above comparative results are placed in Table 4, Figure 8 and Figure 9.

### 4.6. Experiments on Dataset MPMID

**Figure 10:**
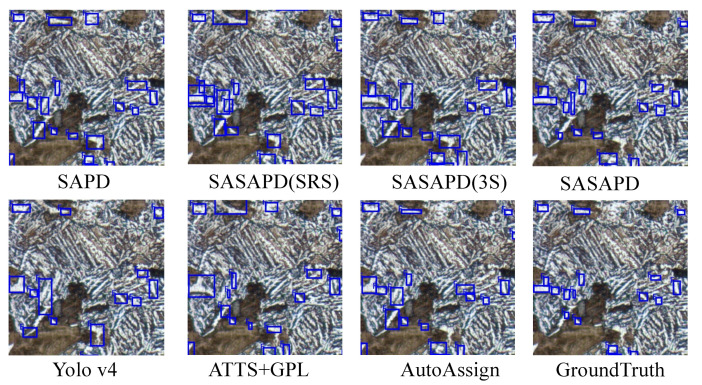
The visual comparison of identifying ferrite (F) on dataset MPMID.

**Figure 11:**
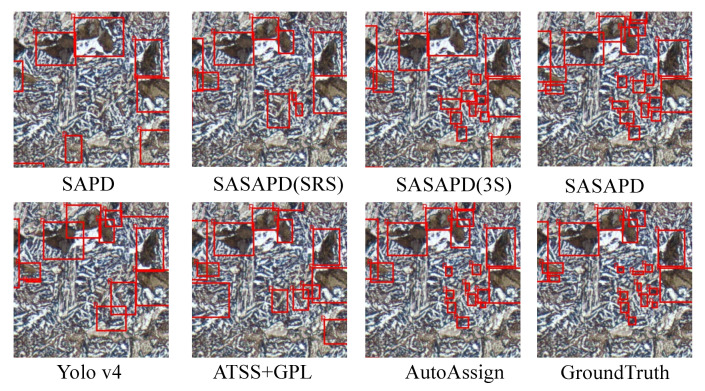
The visual comparison of identifying pearlite (P) on dataset MPMID.

**Figure 12:**
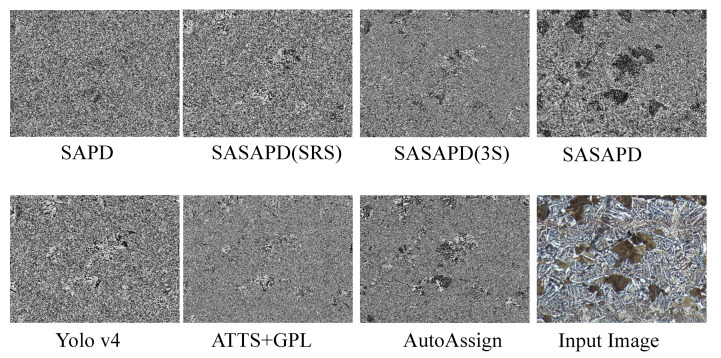
The visual comparison of the feature maps obtained by different models.

In this section, we report ablation study and user study against other outstanding models. To demonstrate effectiveness of the three improvements mentioned in Section 3.3, we design the following experiments as our ablation studies. (1) SAPD: As a baseline, it is trained with the settings reported in work [30]. (2) SASAPD(SRS): It uses SRS to prevent attention bias while keeps the rest unchanged. (3) SASAPD(3S): Based on SASAPD(SRS), it adopts self-adaptive strategy to assign different contributions of pyramid features. In order to evaluate the performance, we further compare SASAPD with other state-of-the-art detectors on dataset MPMID, including SAPD [30], AutoAssign [31], YoloV4 [14] and ATTS+GFL [50]. For a fair comparison, all the models except YoloV4 are equipped with backbone network ResNeXt-101 that proves effective in most cases. Besides, YoloV4 considers EfficientNet-B3 as the backbone network. The detection results on MPMID are present in Table 5, Figure 10 and Figure 11. In addition, we also output the feature maps of the comparative models in Figure 12. Detailedly, for SAPD, ATTS+GPL and SASAPD, we present the feature maps before detection head. YoloV4 visualizes the output of Neck network, and AutoAssign outputs the features of confidence map.

## 5. Results and Discussion

### 5.1. Analysis of Segmentation Results on Dataset SPMID

In this section, we undertake discussions about the ablation study and user study on dataset SPMID.

#### 5.1.1. Discussion about Ablation Study on Dataset SPMID

We present the results of ablation study in Table 4. From the results, it can be observed MAUNet(Dual) consistently outperforms U-Net on all metrics. This improvement is attributed to the dual-path attention. Using module FAE, MAUNet- increases the IoU by up to 0.228 points, which meets the expectations for the design in Section 3.1. With the help of common-used tricks, MAUNet could achieve slightly better performance than MAUNet-. Besides, we take a closer look at the ablation study in the first row of Figure 8. From empirical observation, MAUNet(Dual) could focus on the location of carbide when compared with U-Net. After introducing FAE module, we find that MAUNet- is more successful in fine detection of edges or the shape of carbide. Furthermore, the removal of overlapping strategy and other tricks used in MAUNet aids in refining the details of carbide, providing a closer segmentation result to the ground-truth. Therefore, we can safely draw the conclusion that our model offers more accurate results than other methods quantitatively and qualitatively.

#### 5.1.2. Discussion about User Study on Dataset SPMID

The quantitative analyses from all the test cases are reported in Table 4. As can be seen, our method MAUNet significantly outperforms all the comparative methods in terms of metrics Dice and IoU. Compared with the second best method(mU-Net), MAUNet achieves the Dice value increased by 2.17% and the IoU value increased by 4.18%. As for RoC, MAUNet is closer to the ground-truth RoC (1.25). Additionally, benefiting from complex structures and attention module, the other comparative methods (SAUNet, UNet++, ANU-Net, mU-Net, Deeplab v3+) always perform better than U-Net on these three performance metrics.

In Figure 8, we visually present the segmentation results of different methods on Dataset SPMID. It can be seen that U-Net and ANU-Net cause too many fragments with lower accuracy. Since dense skip connections are utilized, it appears that UNet++ fails to produce clear and pleasing segmentation because of outliers and noises. Also, we observe that mU-Net is able to reject those outliers and display finer results. This is due to the fact that adaptive filter could prevent duplication of low-resolution feature that does harm to the clear texture. In Figure 9, we can see that the response of high-frequency map could describe the edges and textures better, which verifies the effectiveness of high-frequency extraction procedure in skip connection.

Apart from the superior to the competing models, we also evaluate the parameters and running speed and place the results in the last two columns of Table 4. All models are improved based on conventional U-Net, which bring additional parameters and longer running time, but they are all in the same scale. Note that MAUNet elapses less time than MAUNet- even with more layers. The phenomenon is explained by the removal of overlap strategy that needs additional cost. For Deeplab V3+ model, it takes almost three days for training on our device, and requires more than 10 times longer than U-Net. Therefore, we observe that our model achieves comparable performance in terms of parameters and running time, which is appropriate for the devices with limited computation resources.

### 5.2. Analysis of Detection Results on Dataset MPMID

In this section, we make discussions about ablation study and user study on dataset MPMID.

#### 5.2.1. Discussion about Ablation Study on Dataset MPMID

As summarized in the top half of Table 5, comparable results are achieved to verify the effectiveness of each component of the proposed SASAPD. When compared with SAPD, 6.7% Dice, 5.9% Precision, and 7.4% Recall values are gained by SASAPD to identify pearlite (P). The role of SRS puts emphasis on positive samples, which facilitates the improvements on metrics Precision(P) and Precision(F). Next, we study the effect of 3S strategy and apply it to SASAPD(SRS). As long as each instance is assigned to more pyramid levels with self-adaptive weights, we find that SASAPD(3S) obtains 2.23% Dice, 1.33% Precision and 3.07% Recall improvements over SASAPD(SRS) while detecting pearlite (P). To analyze the design of ALFP, we compare SASAPD(SRS) with SASAPD and report the result in Table 5. Since ALFP tends to perceive smaller objects, we find that it brings more improvements on the detection result of ferrite (F). Besides, we display the visual results of ablation study Figure 10 and Figure 11. SASAPD(SRS) is good at recognizing positive objects in larger size, and SASAPD(3S) is able to find more objects with the help of weighted pyramid features. However, the detection result of F obtained by SASAPD(3S) illustrated in Figure 11 has uncertain and inaccurate bounding boxes.

#### 5.2.2. Discussion about User Study on Dataset MPMID

We present the comparative results over several state-of-the-art detection methods in the bottom half of Table 5. Our proposed SASAPD has clear advantages over all competing methods on the tasks of detecting P and F. After joint representation of localization quality estimation and classification estimation, ATSS+GPL improves the baseline SAPD by absolute 3.45% Dice, 2.44% Precision and 4.43% Recall when detecting P. As a representative one-stage anchor-based detector, Yolov4 combines universal features including Weighted-Residual-Connections (WRC), Cross-Stage-Partial-Connections (CSP), Self-Adversarial-Training (SAT) and Cross-mini-Batch Normalization (CmBN), achieving major improvements on all metrics compared with SASAPD. Moreover, benefiting from the automatic assign strategy of determining positive/negative samples, AutoAssign achieves consistent improvement to all existing methods except SASAPD. Owing to the score hypothesis for each anchor point, in comparison with the second best method AutoAssign, our SASAPD gains 1.2%, 0.9% and 1.7% performance on metrics Dice, Precision and Recall when detecting F. Now, let’s take a look at all the results in terms of Recall (grain size). The more the grain size is, the better the detection method is. We can see our proposed SASAPD outperforms the other comparative methods. At the time of inference, the inference speed is measured by Frames-per-Second (FPS). Thanks to the lightweight module, we observe that the running speeds of all the listed modes are close.

In order to understand the performance of all the models better, we demonstrate some detection results of P and F in Figure 10 and Figure 11. By introducing attention mechanism into pyramid level, SASAPD generates few false negatives as well as false positives for both P and F. ATTS+GPL suffers from false negative samples despite that it assigns different weights based on the location quality. The result of YoloV4 is affected by the detection error of smaller objects. We speculate that it mainly because the fixed weights are adopted in Spatial Pyramid Pooling(SPP). We also compare our SASAPD with AutoAssign, which also designs a reweighing strategy to boost detection performance. However, we see that the center weighting proposed in AutoAssign fail to recognize overlapped objects and false positive samples with similar appearances and shapes. Besides, we make a brief discussion about the discriminable ability of the features obtained by different models. From the results in Figure 12, it can be observed that SASAPD shows more discriminable features for identifying microstructure instance than the others. Therefore, it can safely come to the conclusion that SASAPD yields the closest results to the ground-truth in this experiment.

## 6. Conclusions and Future Work

In this paper, we have established two attention-aware deep neural networks (MAUNet and SASAPD) to analyze metallographic images. For the case of SPMID, MAUNet rebuilds the encoder and skip connection by processing high-frequency and low-frequency information independently, and reconstructs the decoder by using dual-path attention blocks. For the case of MPMID, SASAPD is proposed to detect different constitution in an anchor-free way. It adopts SRS strategy to prevent attention bias, and designs a soft self-adaptive selection strategy for the attention-aware pyramid-levels to perceive smaller objects. Extensive experiments have been conducted on self-proposed datasets and detailed analysis are reported on issues such as the effectiveness of each key components, and the computation cost. When applied to dataset SPMID, MAUNet increases Dice and IoU by 22.20% and 42.67% compared with baseline U-Net. When applied to dataset MPMID, SASAPD gains 10.12%, 8.68% and 11.54% performance on metrics Dice, Precision and Recall in comparison with the baseline SAPD when detecting F. In terms of computation cost and running time, these two models can be equipped in the devices with lower computation resources. These experiments, together with a carefully designed user study, consistently validate the effectiveness and robustness of our models in comparison to the state-of-the-art.

However, there still remain the following limitations and possible directions of our work. (1) The number of stages and pyramid levels are determined by experience. The two parameters in our manuscript are set as the same as those in UNet and SAPD, which play significant roles in network design. Though our models with the mentioned settings in Section 4.4 achieve better performance, it is still a challenging task to design a reasonable network automatically. Now, we try to address it with the technology of network architecture search (NAS). (2) The initialization of pyramid levels is essential. At the first 6 epochs, each instance is assigned to the pyramid level which has the minimal loss to stabilize the training model. Namely, the performance of our model heavily relies on the initialization results. In our future work, we are working to get rid of the initialization procedure.

## Figures and Tables

**Table 1:** A summary of the related methods.

Ref.	Proposed	Finding	Limitation
[7]	3D CNN	proposes 3DCNN to extract microstructural.	The computation cost is too much.
[20]	FCN	adapts contemporary classification networks (AlexNet and GoogLeNet) into FCNs.	needs extra fine-tuning layer for postprocessing.
[21]	DeepLab	utilizes atrous spatial pooling and multi-scale atrous pyramid features with encoder-decoder.	The computation cost is too much.
[22]	U-Net	use a contracting path to capture context and a symmetric path that enables precise localization.	High-frequency information in skip connection is lost.
[23]	U-Net Based GCN	adapts a per-pixel feedback to the generator and a per-pixel consistency regularization technique.	High-frequency information in skip connection is lost.
[24]	BCDU-Net	U-Net included BConvLSTM and inserts a densely connected convolutional block.	dense layer brings too much computation cost.
[25]	Mask-RCNN	uses Mask RNN for instance segmentation with different loss functions.	complex generation procedure of candidate generation.
[14]	Yolov4	applies some tricks on Yolov3.	The heavily dependent on pre-defined proposals; Poor performance for tiny objects.
[26]	TridentNet	constructs a parallel multi-branch architecture where each branch shares the same parameters.	treats all the scales equally.
[27]	Cornernet	reformulates the detection problem as locating several key points of the bounding boxes.	The corner points still models a bounding box.
[28]	ExtremeNet	locate the extreme points of objects with supervision from ground-truth mask annotation.	relies on handcrafted clustering to compose whole objects.
[29]	FCOS	regresses the four sides from the center points to form the final bounding box outputs.	Better performance comes at a high computation cost.
[18]	FSAF	applies online feature selection to train anchor-free branches in the feature pyramid.	only selects the optimal feature level for each instance.
[30]	SAPD	assigns optimal feature levels to given sample based on the loss distribution in object detection.	fails to obtain discriminable features with poor sample weighting.
[31]	AutoAssign	determines positive/negative samples by generating proper weights to modify each location’s prediction.	fails to output satisfying results when the objects are with similar appearances and shapes.

**Table 2:** The data distribution of dataset Single-Phase Metallographic Image Dataset (SPMID) based on carbon level and segregation degree.

	Carbon	A	B	C	D	E
Segregation						
1		345	432	341	456	298
2		353	451	357	419	370
3		367	386	394	373	451
4		346	401	410	402	269
5		296	447	391	323	355

**Table 3:** The data distribution of dataset Multi-Phase Metallographic Image Dataset (MPMID). The first row is the number of pixels, and the second row is the proportion.

F	P	Segband	Up B	Background	Total
632,880	369,920	68,560	25,200	417,520	1,514,080
41.80%	24.43%	4.53%	1.66%	27.58%	-

**Table 4:** The quantitative comparison of the segmentation results on dataset SPMID. The best results are highlighted in bold.

Model	Dice	IoU	RoC	Params	Runing Time (s)
U-Net	0.786	0.645	0.981	**7.8 M**	**4.86**
MAUNet (Dual)	0.836	0.715	1.079	7.9 M	4.92
MAUNet-	0.934	0.873	1.260	8.2 M	5.17
SAUNet	0.831	0.711	1.037	9.3 M	4.99
UNet++	0.875	0.777	1.264	9.0 M	8.73
ANU-Net	0.906	0.828	1.149	8.9 M	6.42
mU-Net	0.940	0.886	1.257	8.5 M	5.25
Deeplab V3+	0.793	0.652	1.037	352.5 M	16.76
MAUNet(Ours)	**0.963**	**0.923**	**1.257**	8.8 M	5.02

**Table 5:** The quantitative comparison of identifying the elements of ferrite (F) and pearlite (P) on dataset MPMID. The best results are highlighted in bold.

Input	Backbone	The Number of Phases	Anchor Free or not	Dice (F)	Precision (F)	Recall (F)	Dice (P)	Precision (P)	Recall (P)	FPS
SAPD(SRS)	X-101-32x4d-DCN	one	yes	0.918	0.928	0.908	0.911	0.943	0.881	25
SAPD(3S)	X-101-32x4d-DCN	one	yes	0.932	0.942	0.921	0.931	0.956	0.905	22
SAPD	X-101-32x4d-DCN	one	yes	0.876	0.893	0.857	0.887	0.913	0.865	28
SASAPD	X-101-32x4d-DCN	one	yes	**0.963**	**0.971**	**0.954**	**0.947**	**0.967**	**0.928**	20
AutoAssign	X-101-32x4d-DCN	one	yes	0.951	0.964	0.938	0.937	0.958	0.914	20
Yolo V4	EfficientNet-B3	one	no	0.943	0.953	0.930	0.931	0.951	0.911	**31**
ATSS+GFL	X-101-32x4d-DCN	one	yes	0.914	0.934	0.895	0.918	0.936	0.903	18

## Data Availability

The data presented in this study are openly available in github project.
